# CNS Transduction Benefits of AAV-PHP.eB over AAV9 Are Dependent on Administration Route and Mouse Strain

**DOI:** 10.1016/j.omtm.2020.10.011

**Published:** 2020-10-20

**Authors:** Sophie N. Mathiesen, Jasmine L. Lock, Lucia Schoderboeck, Wickliffe C. Abraham, Stephanie M. Hughes

**Affiliations:** 1 Department of Biochemistry, Brain Health Research Centre, Brain Research New Zealand–Rangahau Roro Aotearoa, University of Otago, Dunedin 9016, New Zealand; 2 Department of Psychology, Brain Health Research Centre, Brain Research New Zealand–Rangahau Roro Aotearoa, University of Otago, Dunedin 9016, New Zealand

**Keywords:** viral vector, AAV, AAV9, AAV-PHP.eB, gene therapy, central nervous system, intravenous

## Abstract

Adeno-associated viral (AAV) vectors are attractive tools for central nervous system (CNS) gene therapy because some vectors can cross the blood-brain barrier (BBB), allowing them to be used as minimally invasive treatments. A novel AAV vector recently evolved *in vivo*, AAV-PHP.eB, has been reported to cross the BBB more effectively than the existing gold standard AAV9, but not under all conditions. Here, we compared the efficacy of single-stranded AAV-PHP.eB and AAV9 in targeting mouse CNS and peripheral tissues after administration via various routes, in two different mouse strains (C57BL/6J and B6C3), and after packaging AAV-PHP.eB with a self-complementary genome. We found that AAV-PHP.eB produced higher CNS transduction than AAV9 after intravenous injection, but only in C57BL/6J and not in B6C3 mice. AAV-PHP.eB and AAV9 produced similar CNS transduction when the administration route did not require the vectors to cross the BBB. Packaging AAV-PHP.eB with a self-complementary genome increased overall CNS transduction, but at the expense of strong neuronal tropism. AAV-PHP.eB resulted in less transduction of liver tissue than AAV9 under all conditions. Taken together, these results suggest the potential for AAV-PHP.eB as a vector for CNS gene therapy applications, but consideration will be required for translation beyond mouse models.

## Graphical Abstract

**Figure:**
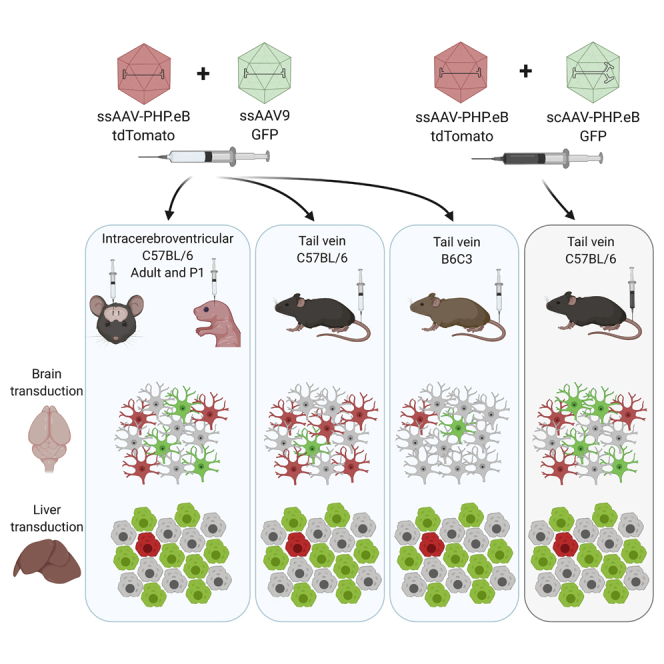

## Teaser

Engineered AAV vector AAV-PHP.eB has improved ability over AAV9 to transduce mouse CNS after intravenous administration. Using various methods of administration, we show this is due to the increased ability of AAV-PHP.eB to cross the blood-brain barrier; however, this is dependent on the mouse strain used.

## Introduction

One of the biggest hurdles in the treatment of neurological diseases is finding effective methods to deliver therapeutics to the central nervous system (CNS). Recombinant adeno-associated viral (rAAV) vectors have long been one of the most favored gene therapy vectors for CNS disease applications because of their ability to give stable, long-lasting transgene expression in non-dividing cells.^1^ From a clinical standpoint, therapeutics that can be given by a non-invasive systemic route will be crucial for translation to humans on a large-scale basis. However, naturally occurring AAV capsid serotypes have limited ability to cross the blood-brain barrier (BBB). This means they must either be given in very high doses intravenously (i.v.) or be administered by local injection into the CNS, making them inappropriate for use as a non-invasive treatment approach.

Multiple techniques for increasing the efficacy of rAAV gene transfer to the brain from the bloodstream have been investigated to overcome this issue, including increasing BBB permeability with pharmacological agents or ultrasound,^2^ and using shuttle peptides to increase the ability of the capsid to cross the BBB.^3^ Arguably one of the most successful strategies has been the *in vivo*-directed evolution of the AAV9 capsid by the Cre Recombinase-based Targeted Evolution (CREATE) system. The CREATE system produced the novel AAV-PHP.B capsid family (including AAV-PHP.B, and the second generation, AAV-PHP.eB), which differed from AAV9 by a heptamer amino acid insertion in the capsid sequence.^4^^,^^5^ These capsids were generated by a Cre recombinase system that selected for capsids that were highly efficient at transducing the CNS in adult C57BL/6J mice after administration by i.v. injection, with AAV-PHP.eB giving upward of a 50-fold increase in cell transduction in multiple brain regions.^4^

Although AAV-PHP.B vectors seemed promising for broadening systemic gene therapeutics, further characterization work soon revealed that their increased BBB permeability did not extend to non-human primates,^6^^,^^7^ and not even to all strains of mice.^8^ Efforts to elucidate the reason behind these capsids being “CNS permissive” in some animals, but not others, demonstrated that the capsid modification led to interactions with the LY6A (SCA-1) protein expressed on brain microvascular endothelial cells (BMVECs) in the BBB; it is likely this interaction gives the capsid high shuttling efficiency through the BMVECs.^9^^,^^10^ Unfortunately, in about 50% of mouse strains, the *Ly6a* gene contains single-nucleotide polymorphisms (SNPs) prohibiting the interaction of AAV-PHP.B capsids with LY6A, deeming them “CNS non-permissive” in these animals. Furthermore, there is no LY6A homolog in primates, explaining why AAV-PHP.B is CNS non-permissive in primates, and this has severe implications for translation of these vectors to humans.^11^

Despite these findings, the AAV-PHP.B viral vectors still provide a valuable tool for preclinical testing, provided that tests can be carried out in CNS-permissive animals. In this study, we sought to further characterize the AAV-PHP.eB capsid in terms of its application in a range of preclinical gene therapy approaches. Primarily, we were interested in comparing AAV-PHP.eB with the existing gold standard rAAV serotype, AAV9, by giving both serotypes in a combined injection to eliminate potential variation in administration consistency. To achieve this, we packaged each vector with a differently colored fluorescent protein (green fluorescent protein [GFP] or tdTomato), each controlled by a ubiquitous CAG/CB7 promoter to result in transgene expression in any brain cell types transduced. We investigated the transduction efficiency of the AAV-PHP.eB vector to get a global picture of the vector properties under a range of conditions. This included administration by intracerebroventricular (i.c.v.) injection at two different mouse ages, because there have previously been varied findings in regard to rAAV cell-type transduction and vector spread dependent on mouse age.^12^, ^13^, ^14^ Further, we assessed AAV-PHP.eB transduction by intranasal injection, by i.v. injection in a previously untested strain of mouse (B6C3), and when packaged with a self-complementary (sc) genome. We found that when the need for the vector to cross the BBB was eliminated by the administration method, AAV9 and AAV-PHP.eB performed similarly, reaffirming that it is the ability of AAV-PHP.eB to cross the BBB that increases its CNS transduction efficiency. We also demonstrated that B6C3 mice are CNS non-permissive to AAV-PHP.eB, and that a scAAV genome increases overall transduction efficiency but at the expense of neuronal transduction.

## Results

### AAV9 and AAV-PHP.eB Produce Widespread Transgene Expression in the Brain after i.c.v. Delivery in Post-natal Day (P) 1 and Adult Mice

**Figure 1:**
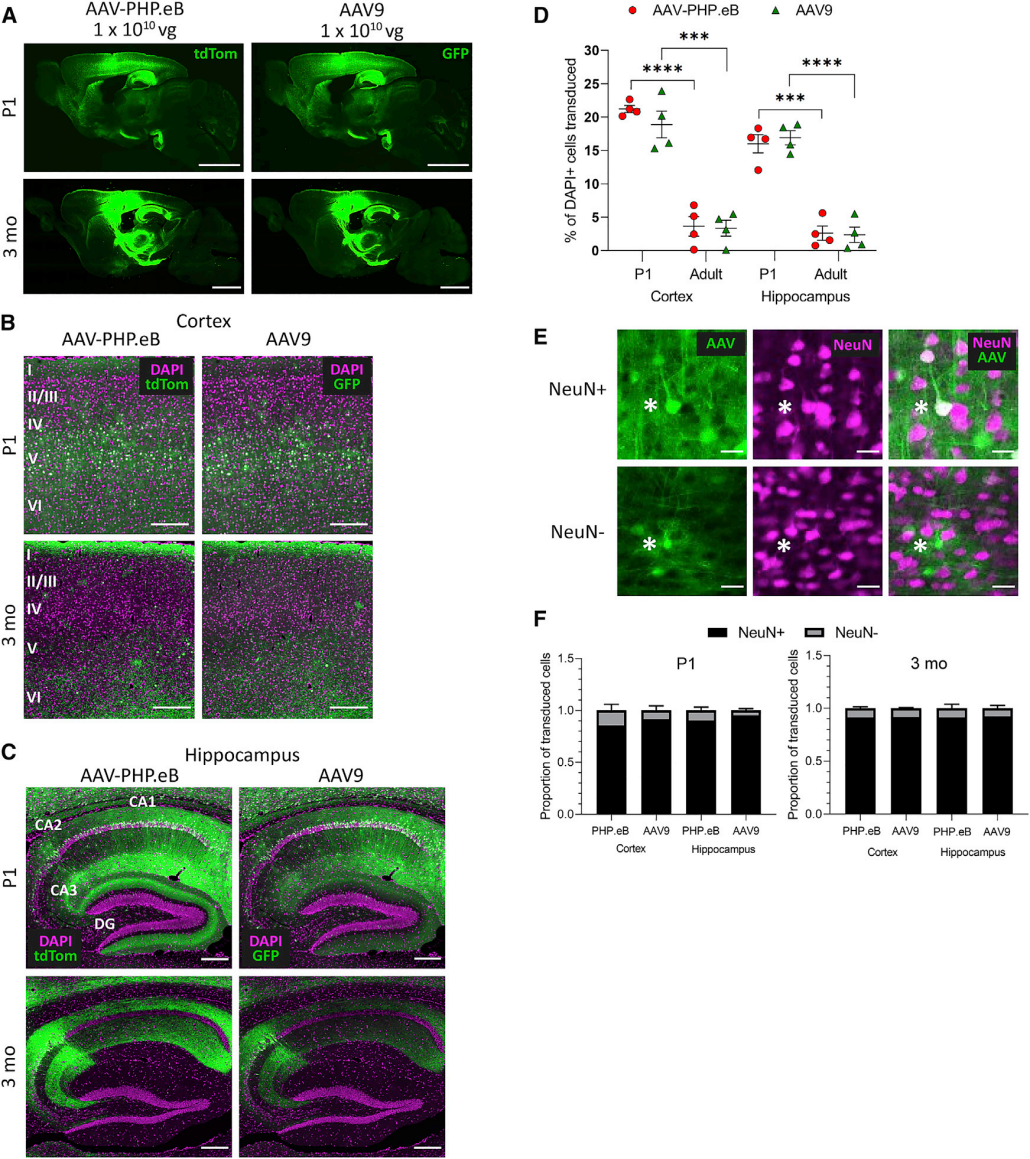
AAV-PHP.eB and AAV9 Give Similar Cell Transduction When Administered via Intracerebroventricular (i.c.v.) Injection (A) Representative whole-brain images of native tdTomato (PHP.eB) and GFP (AAV9) fluorescence after 30 days of expression. (B and C) Representative images of native transgene fluorescence (green) and DAPI staining (magenta) in the somatosensory cortex (B) and hippocampus (C). (D) The percentage of DAPI^+^ cells that were transduced by AAV-PHP.eB or AAV9 in the somatosensory cortex and hippocampus in P1 and 3-month-old mice. (E) Representative images of a NeuN^+^ and NeuN^−^ cells transduced by AAV (cells marked with asterisk). (F) The proportion of NeuN^+^ versus NeuN^−^ cells transduced by AAV-PHP.eB or AAV9 in the cortex and hippocampus in P1 and 3-month-old animals. For quantification: n = 4 mice per group, mean ± SEM; unpaired two-tailed t test (∗∗∗∗p < 0.0001, ∗∗∗p < 0.001). Scale bars: 2,000 μm (A); 200 μm (B and C); 20 μm (E). DG, dentate gyrus; GFP, green fluorescent protein; PHP.eB, AAV-PHP.eB; tdTom, tdTomato.

Although some neurological diseases require gene therapy treatments to cover a vast number of cells spread across the brain, other diseases may benefit from a targeted treatment localized to one or few brain regions. It is therefore of interest to know whether the AAV-PHP.eB serotype has comparable efficacy to AAV9 when injected locally into the cerebrospinal fluid (CSF) and whether the animal age at injection impacts on transduction or spread. After i.c.v. injection into P1 pups, we found widespread cell transduction from both AAV9 and AAV-PHP.eB vectors (Figure 1A), including along the dorsal cortex, striatum, hippocampus, and thalamus. In particular, there was strong expression of GFP and tdTomato through the cortex layer V (Figure 1B) and the hippocampal regions Cornu Ammonis 2 (CA2) and subiculum (Figure 1C). After i.c.v. injection into adult mice, we found a similar widespread cell transduction in regions surrounding the lateral ventricles, including cortex, hippocampus, and thalamus (Figures 1B and 1C). In comparison with P1 animals, neither serotype spread as far through the cortex or subcortical regions in the adult brain. Cell counts expressed as a percentage of DAPI-positive (DAPI^+^) cells revealed no significant differences in transduction efficiency between the two serotypes dependent on brain region or animal age, but both serotypes transduced significantly more DAPI^+^ cells in P1 than in adult animals in both the cortex (AAV-PHP.eB: P1, 21.2% ± 1.0% versus 3 months, 3.6% ± 2.9%, t(6) = 11.2, p < 0.0001; AAV9: P1, 18.9% ± 3.9% versus 3 months, 3.4% ± 2.4%, t(6) = 6.7, p = 0.0005) and the hippocampus (AAV-PHP.eB: P1, 16.0% ± 2.7% versus 3 months, 2.6% ± 2.1%, t(6) = 7.7, p = 0.0002; AAV9: P1, 16.9% ± 2.1% versus 3 months, 2.4% ± 2.3%, t(6) = 9.3, p < 0.0001).

### AAV9 and AAV-PHP.eB Have Strong Neuronal Tropism after i.c.v. Injection

To identify the proportion of neuronal cells transduced by each serotype, we used an antibody directed against neuronal nuclear protein (NeuN) to detect neurons in our regions of interest. Given that almost all mature neurons *in vivo* in the hippocampus and cortex can be positively labeled with a NeuN antibody (NeuN^+^),^15^ it was assumed that any transduced cells that were NeuN negative (NeuN^−^) were not neurons. Figure 1E gives an example of a NeuN^+^ cell compared with a NeuN^−^ cell.

In P1 animals, both AAV-PHP.eB and AAV9 transduced a larger proportion of NeuN^+^ than NeuN^−^ cells in the cortex and the hippocampus (Figure 1F). Similarly, in adult animals, AAV-PHP.eB and AAV9 transduced a significantly larger proportion of NeuN^+^ than NeuN^−^ cells in the cortex and the hippocampus (Figure 1F). There were no differences in the proportions of NeuN^+^ cell transduction between viruses at either animal age.

### AAV-PHP.eB Has Similar Transduction Efficacy to AAV9 via Intranasal Injection

**Figure 2:**
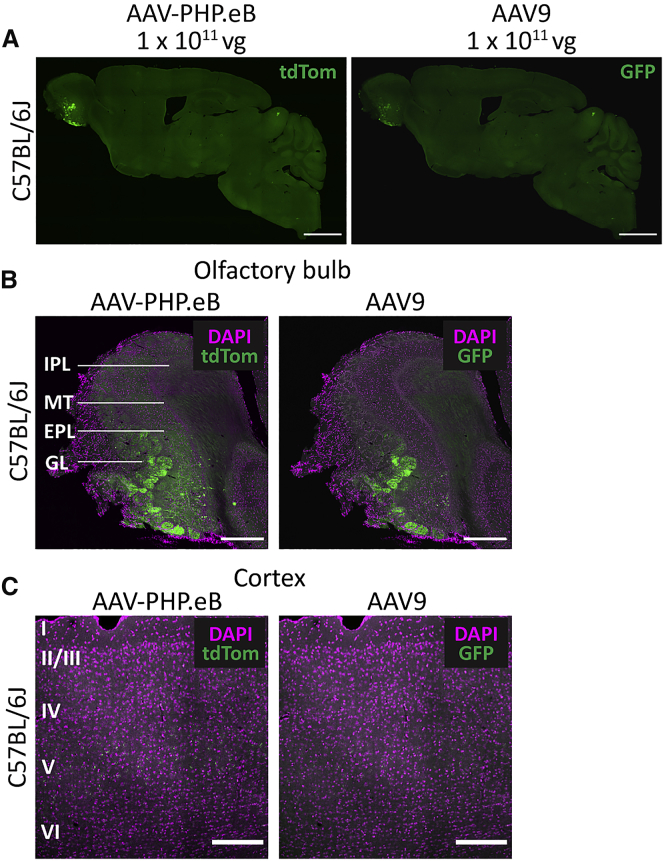
AAV-PHP.eB Produces Similar Cell Transduction to AAV9 after Intranasal Injection (A) Representative whole-brain images of native tdTom (AAV-PHP.eB) and GFP (AAV9) transgene fluorescence after 30 days of expression. (B and C) Representative images of native fluorescence (green) and DAPI staining (magenta) in the olfactory bulb (B) and cortex (C). Scale bars: 2,000 μm (A); 200 μm (B); 500 μm (C). EPL, external plexiform layer; GL, glomerular layer; IPL, inner plexiform layer; MT, mitral/tufted cell layer.

Because non-invasive systemic treatments are the primary goal for modified viral vectors, we next wanted to assess the transduction efficiency of AAV-PHP.eB compared with AAV9 by a non-invasive administration route, intranasal injection. It was of interest to see whether the increased transduction capability of AAV-PHP.eB was also apparent after intranasal injection, because it is thought that the intranasal pathway bypasses the BBB.^16^ Intranasal injection gave limited transgene expression from both AAV-PHP.eB and AAV9, which was present only in the olfactory bulb (Figure 2A). This transgene expression appeared to be predominately located in neuronal projections entering the olfactory glomeruli (Figure 2B). There was no cell transduction by either vector in regions posterior to the olfactory bulb, such as the cortex (Figure 2C).

### AAV-PHP.eB Has Higher Transduction Efficacy Than AAV9 after i.v. Injection in C57BL/6J Mice

**Figure 3:**
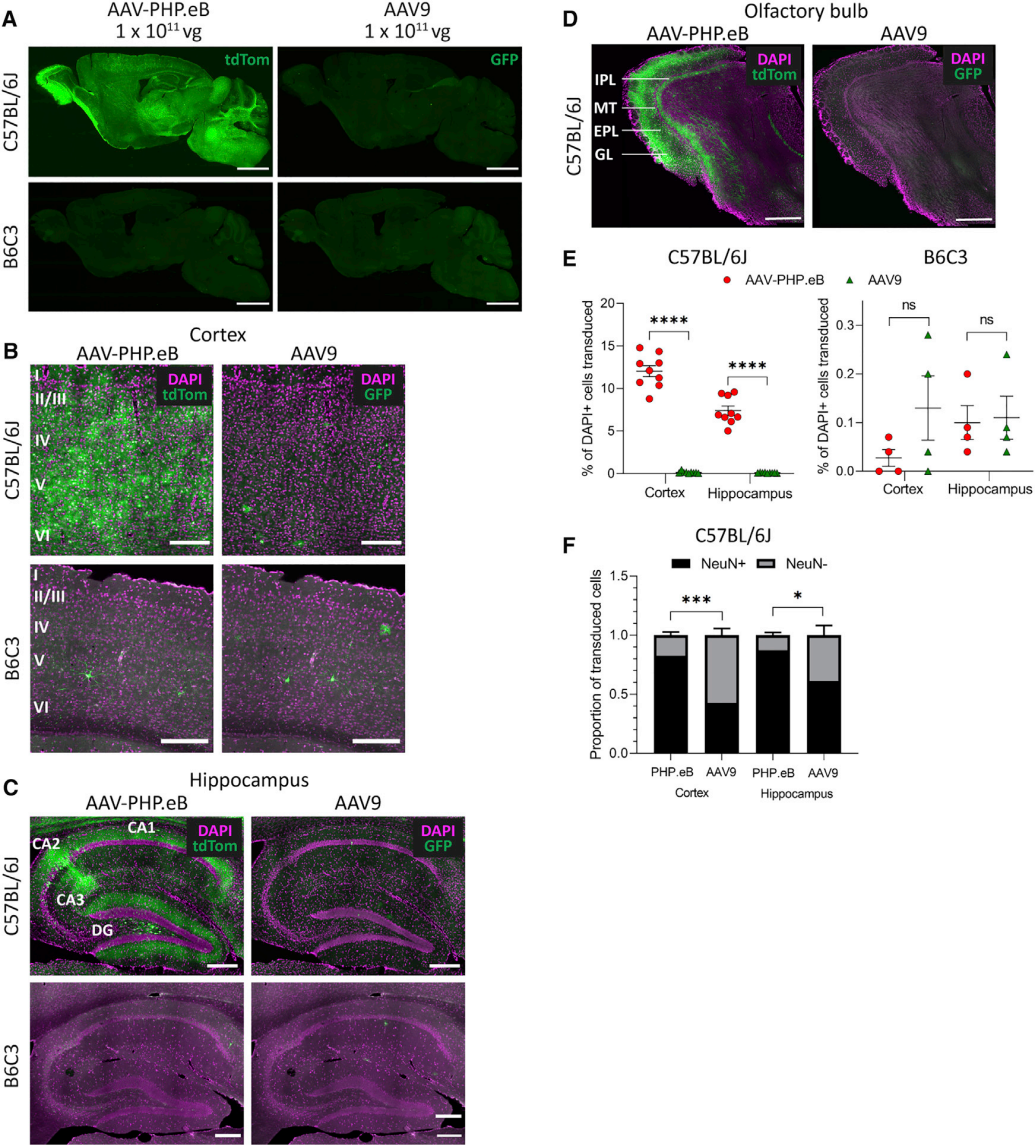
AAV-PHP.eB Gives Higher Cell Transduction Than AAV9 in C57BL/6J, but Not B6C3, Mice after Intravenous (i.v.) Injection (A) Representative whole-brain images of native tdTom (PHP.eB) and GFP (AAV9) fluorescence after 30 days of expression. (B–D) Representative images of native transgene fluorescence (green) and DAPI staining (magenta) in the somatosensory cortex (B), hippocampus (C), and olfactory bulb (D). (E) The percentage of DAPI^+^ cells that were transduced by AAV-PHP.eB or AAV9 in the cortex and hippocampus of C57BL/6J and B6C3 mice. (F) The proportion of NeuN^+^ versus NeuN^−^ cells transduced by AAV-PHP.eB or AAV9 in the cortex and hippocampus in C57BL/6J mice. For quantification: n = 9 mice in the C57BL/6J group, n = 4 mice in the B6C3 group, mean ± SEM; paired two-tailed t tests (∗∗∗∗p < 0.0001, ∗∗∗p < 0.001, ∗p < 0.05). Scale bars: 2,000 μm (A); 200 μm (B and C); 500 μm (D).

To validate previous findings, we then injected AAV9 and AAV-PHP.eB into the lateral tail vein of adult C57BL/6J mice. Transduction with AAV9 was minimal because there was very little GFP expression anywhere in the brain (Figures 3A–3D). Conversely, transduction of all brain areas with AAV-PHP.eB could be seen by tdTomato expression, including across the cortex (Figure 3B), with higher transduction in cells of layers IV–V and hippocampus (Figure 3C), particularly subregion CA2. There was also strong transduction in thalamus, cerebellum, brainstem and olfactory bulb glomerular, external plexiform, and internal plexiform layers (Figures 3A and 3D).

AAV-PHP.eB transduced significantly more DAPI^+^ cells than AAV9 in the cortex (12.0% ± 1.9% versus 0.2% ± 0.1% DAPI^+^ cells, paired t(8) = 18.6; p < 0.0001) and the hippocampus (7.4% ± 1.6% versus 0.10% ± 0.03% DAPI^+^ cells, paired t(8) = 13.6; p < 0.0001) (Figure 3E). AAV-PHP.eB also transduced a significantly larger proportion of NeuN^+^ cells than AAV9, in both the cortex (0.83 ± 0.08 versus 0.43 ± 0.17, paired t(8) = 6.5; p = 0.0002) and the hippocampus (0.87 ± 0.07 versus 0.61 ± 0.25, paired t(8) = 3.1; p = 0.014) (Figure 3F).

### AAV-PHP.eB Is Not CNS Permissive in B6C3 Mice

Because previous studies had indicated that AAV-PHP.eB is not CNS permissive in all mouse strains, we wanted to also assess the efficacy of AAV-PHP.eB compared with AAV9 in the B6C3 strain of mouse. This strain is commonly used as a background for transgenic disease models and had not previously been tested for AAV-PHP.eB CNS permissiveness. We therefore injected 1 × 10^11^ vector genomes (vg) each of single-stranded AAV9 (ssAAV9)-CB7-EGFP and ssAAV-PHP.eB-CAG-tdTomato into the lateral tail vein of adult B6C3 mice, and analyzed GFP and tdTomato expression in the brain 30 days post-injection. Unlike in the C57BL/6J mice, both AAV9 and AAV-PHP.eB transduced very few cells across the brain (Figures 3A–3D), and AAV-PHP.eB was no more effective at transducing the brain than AAV9 in the cortex or the hippocampus (Figure 3E).

### scAAV-PHP.eB Has Higher Overall Transduction Efficiency Than ssAAV-PHP.eB

Another strategy for increasing transgene expression from viral vectors is by packaging the vectors with an sc genome, which removes the rate-limiting conversion of the ssAAV genome to double-stranded DNA^17^ and has been shown to increase cell transduction when applied to the AAV-PHP.B capsid.^18^ Consequently, we investigated whether the systemic efficiency of AAV-PHP.eB could also be improved with a scAAV genome configuration. We therefore i.v. injected ssAAV-PHP.eB and scAAV-PHP.eB in combination into adult C57BL/6J mice.

**Figure 4:**
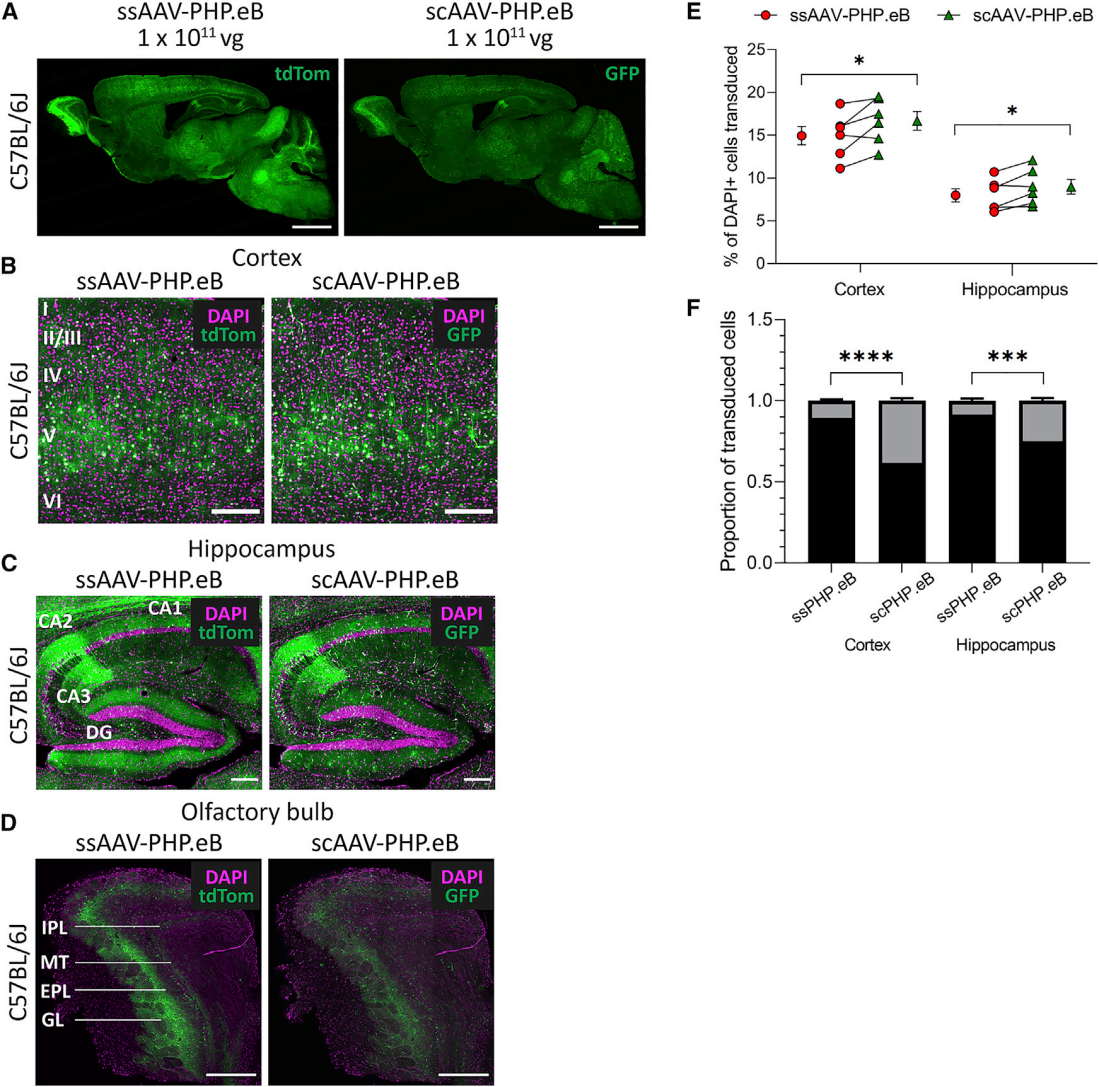
scAAV-PHP.eB Has Higher Transduction Efficiency Than ssAAV-PHP.eB in C57BL/6J Mice after i.v. Injection (A) Representative whole-brain images of native tdTom (ssPHP.eB) and GFP (scPHP.eB) transgene fluorescence after 30 days of expression. (B–D) Representative images of native fluorescence (green) and DAPI staining (magenta) in the somatosensory cortex (B), hippocampus (C), and olfactory bulb (D). (E) The percentage of DAPI^+^ cells that were transduced by ssPHP.eB or scPHP.eB in the cortex and hippocampus. Individual data points show group mean ± SEM. (F) The proportion of NeuN^+^ versus NeuN^−^ cells transduced by ssPHP.eB or scPHP.eB in the cortex and hippocampus. For quantification: n = 6 mice, mean ± SEM; paired two-tailed t tests (∗∗∗∗p < 0.0001, ∗∗∗p < 0.001, ∗p < 0.05). Scale bars: 2,000 μm (A); 200 μm (B and C); 500 μm (D). sc, self-complementary; ss, single-stranded.

The two vectors produced different patterns of transduction dependent on brain region (Figure 4A). Although the two vectors gave similar transduction in the cortex, again concentrated in cortex layer V (Figure 4B), scAAV-PHP.eB gave a modestly higher transduction of cell bodies but less transduction of hippocampal neuropil, particularly around the dentate gyrus, than ssAAV-PHP.eB (Figure 4C). High transduction of the olfactory bulb glomerular and external plexiform layers was also produced by scAAV-PHP.eB, although to a lesser extent than seen with ssAAV-PHP.eB (Figure 4D). There were significantly more DAPI^+^ cells transduced by scAAV-PHP.eB than ssAAV-PHP.eB in the cortex (16.7% ± 2.7% versus 15.0% ± 2.7%; paired t(5) = 2.6; p = 0.049) and the hippocampus (9.0% ± 2.1% versus 8.0% ± 1.9%; paired t(5) = 2.8; p = 0.036) (Figure 4E).

### ssAAV-PHP.eB Has Greater Neuronal Tropism Than scAAV-PHP.eB

Although scAAV-PHP.eB produced transgene expression in a larger number of total cells than ssAAV-PHP.eB, based on the morphology of GFP^+^ cells, it appeared that many were non-neuronal cells. We were therefore interested to see whether there were similar proportions of neuronal versus non-neuronal cells transduced by the two vectors. Of all transduced cells, there was a significantly larger proportion of NeuN^+^ cells transduced by ssAAV-PHP.eB than scAAV-PHP.eB in both the cortex (0.90 ± 0.02 versus 0.62 ± 0.04; paired t(5) = 18.4; p < 0.0001) and hippocampus (0.91 ± 0.03 versus 0.75 ± 0.04; paired t(5) = 7.1; p = 0.0009) (Figure 4F).

### AAV-PHP.eB Has Lower Liver Tropism Than AAV9 after i.c.v. Injection at Two Mouse Ages

Off-target transduction of organs other than the brain is something that must be considered when developing gene therapy treatments for the CNS, because the expression of a given transgene in peripheral organs may be harmful. Vectors given by i.v. administration that are de-targeted for peripheral organs such as the liver will be important for limiting toxicity and maximizing the number of viral particles available to enter the CNS. Given that AAV9 is known to have high liver tropism, we were interested to compare this with liver transduction by AAV-PHP.eB.

**Figure 5:**
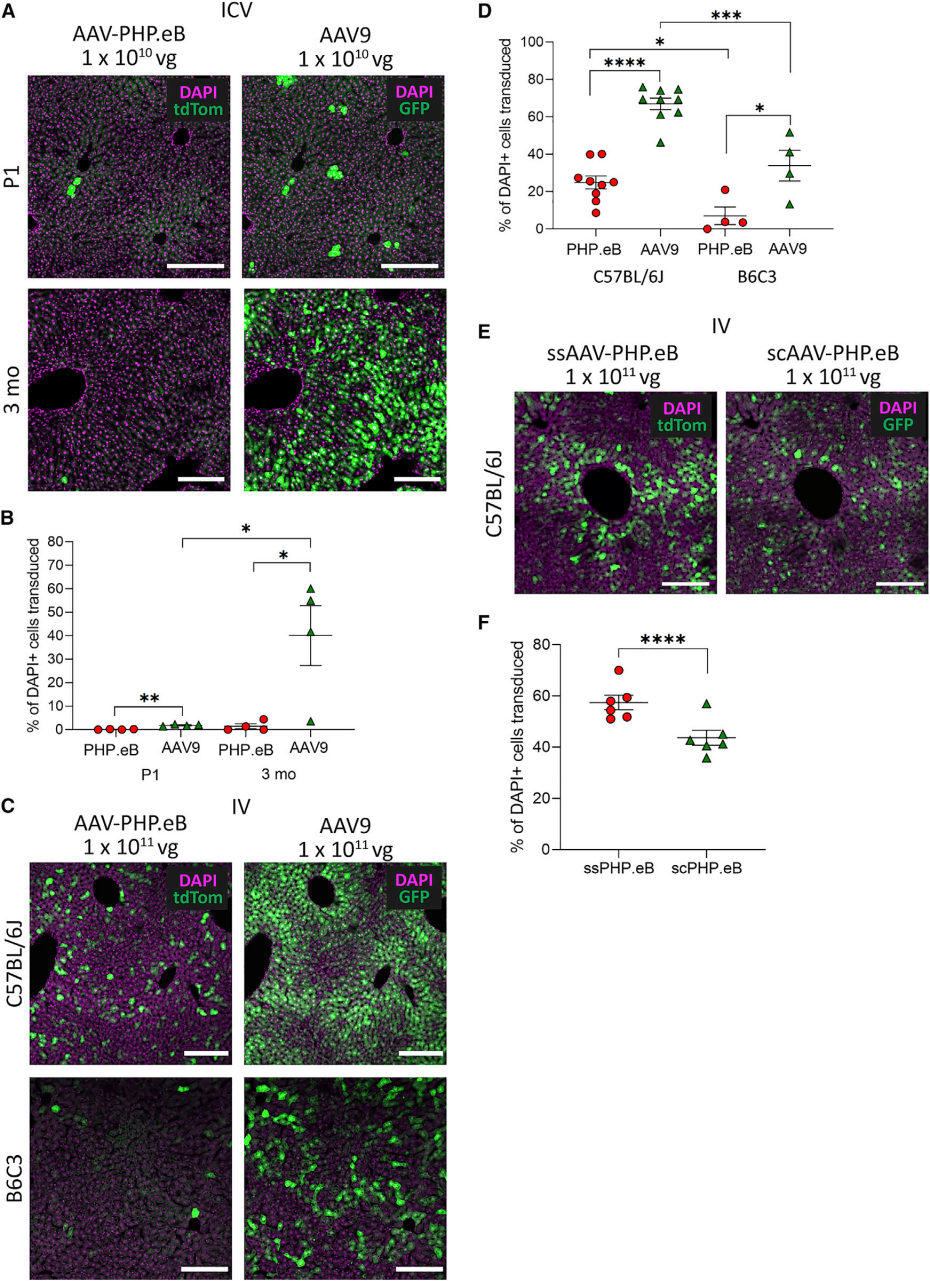
AAV Liver Transduction under Different Administration and Mouse Strain Conditions (A) Representative images of native tdTom (PHP.eB) and GFP (AAV9) transgene fluorescence after 30 days of expression after i.c.v. administration. (B) The percentage of DAPI^+^ liver cells that were transduced by AAV-PHP.eB or AAV9 in P1 and 3-month-old mice. (C) Representative images of native tdTom (PHP.eB) and GFP (AAV9) transgene fluorescence after 30 days of expression after i.v. administration. (D) The percentage of DAPI^+^ liver cells that were transduced by AAV-PHP.eB or AAV9 in C57BL/6J and B6C3 mice. (E) Representative images of native tdTom (ssPHP.eB) and GFP (scPHP.eB) transgene fluorescence after 30 days of expression after i.v. administration in C57BL/6J mice. (F) The percentage of DAPI^+^ liver cells that were transduced by ssAAV-PHP.eB or scAAV-PHP.eB in C57BL/6J mice. For quantification: (B) n = 4 mice per group, mean ± SEM, paired t test (∗∗p < 0.01, ∗p < 0.05); (D) n = 9 C57B/6J mice and n = 4 B6C3 mice, mean ± SEM, paired t test (∗∗∗∗p < 0.0001, ∗∗∗p < 0.001, ∗p < 0.05); (F) n = 6 mice per group, mean ± SEM, paired t test (∗∗∗∗p < 0.0001). Scale bars: 200 μm.

After i.c.v. injection of AAV9 and AAV-PHP.eB in P1 C57BL/6J mice, AAV9 gave significantly higher gene transfer to the liver (1.8% ± 0.4% DAPI^+^ cells) than AAV-PHP.eB (0.12% ± 0.14% DAPI^+^ cells; paired t(3) = 10.3; p = 0.002) (Figures 5A and 5B). In adult mice, i.c.v. injection also resulted in a significantly higher percentage of liver cells being transduced by AAV9 (40.0% ± 25.5% DAPI^+^ cells) than AAV-PHP.eB (1.5% ± 2.1% DAPI^+^ cells; paired t(3) = 3.2; p = 0.049). AAV9 also transduced a significantly larger percentage of liver cells in adult mice than in P1 mice (t(6) = 3.0; p = 0.024), whereas AAV-PHP.eB transduced similar percentages of liver cells regardless of mouse age (t(6) = 1.3; p = 0.25). These findings indicate that there are age-dependent differences in CNS permeability, which mean that viral vectors may leak from the CSF into the periphery, even when injected intracerebrally.

### AAV-PHP.eB Has Lower Liver Tropism Than AAV9 after i.v. Injection in Two Mouse Strains

After i.v. injection of ssAAV9 and ssAAV-PHP.eB in C57BL/6J mice, AAV9 gave significantly higher gene transfer to the liver (66.9% ± 9.3% DAPI^+^ cells) than AAV-PHP.eB (24.9% ± 10.4% DAPI^+^ cells; paired t(8) = 12.8; p < 0.0001) (Figures 5C and 5D). Similarly, in B6C3 mice, AAV9 transduced significantly more liver cells (33.9% ± 16.5% DAPI^+^ cells) than AAV-PHP.eB (7.1% ± 9.4% DAPI^+^ cells; paired t(3) = 4.5; p = 0.02). Both vectors transduced significantly fewer cells in the B6C3 mice than in C57BL/6J mice (AAV-PHP.eB t(11) = 2.9, p = 0.014; AAV9 t(11) = 4.7, p = 0.006).

### ssAAV-PHP.eB Has Higher Liver Tropism Than scAAV-PHP.eB

After i.v. injection of ssAAV-PHP.eB and scAAV-PHP.eB in C57BL/6J mice, ssAAV-PHP.eB produced higher liver cell tropism (57.5% ± 7.0% DAPI^+^ cells) than scAAV-PHP.eB (43.7% ± 7.2% DAPI^+^ cells; paired t(5) = 13.0; p < 0.0001) (Figures 5E and 5F).

### AAV-PHP.eB and AAV9 Produce Limited Heart Transduction after Central and Peripheral Administration

**Figure 6:**
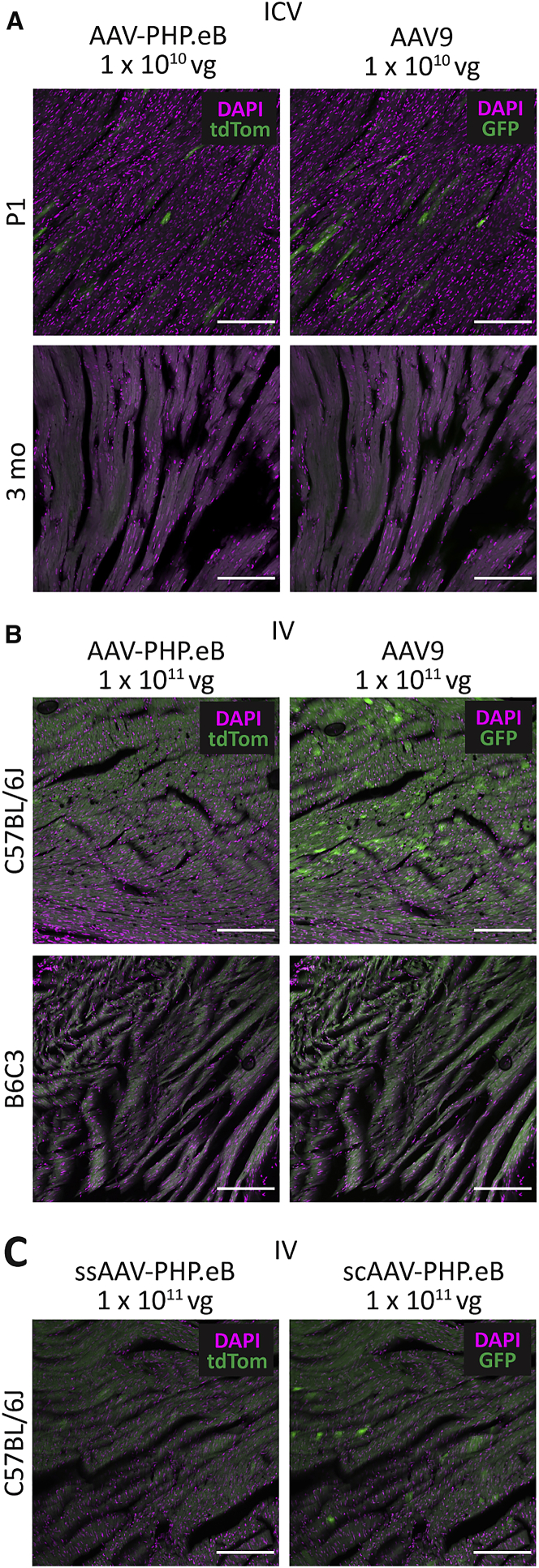
AAV Heart Transduction under Different Administration and Mouse Strain Conditions (A and B) Representative images of native tdTom (AAV-PHP.eB) and GFP (AAV9) fluorescence after 30 days of expression after i.c.v. (A) or i.v. administration (B). (C) Representative images of native tdTom (ssPHP.eB) and GFP (scPHP.eB) transgene fluorescence after 30 days expression after i.v. administration. Scale bars: 200 μm.

As another site of peripheral organ transduction, we examined heart tissue for AAV9 and AAV-PHP.eB transduction and found that there was little heart transduction in P1 mice after i.c.v. injection, again indicating some leakage of the vectors from the CSF into the periphery, although there was no detectable transduction in adult mice after i.c.v. injection (Figure 6A). In addition, there was some transduction by AAV9 in the heart of C57BL/6J mice, but very little by AAV-PHP.eB, and there was no detectable transduction by either vector in B63C mice (Figure 6B). There was slightly more heart transduction by the scAAV-PHP.eB vector as compared with ssAAV-PHP.eB, but transduction was still minimal (Figure 6C).

## Discussion

Here we aimed to assess the CNS and peripheral transduction efficiency of the modified AAV-PHP.eB capsid under administration route, mouse strain, and genomic configurations, and compare it with AAV9, the existing gold standard for CNS gene therapy. A novel aspect of our study was the combination of the viral vectors into a single injection, providing for a within-animal control to account for injection variation.

As anticipated based on previous studies, AAV-PHP.eB produced more effective CNS transduction after systemic injection in C57BL/6J mice but was no more effective than AAV9 when the administration route did not require the vectors to cross the BBB. In the i.c.v. administration condition, AAV-PHP.eB offered no improvement in overall cell transduction or difference in tropism compared with AAV9, in either P1 or adult animals. The higher cell transduction from both vectors in P1 animals may be a consequence of improved spread of the virus throughout the extracellular space of the brain, because the transduction in P1 brain was sparser and more widely distributed than the relatively concentrated transduction in regions around the ventricle in the adult brain. A difference in transduction in P1 mice is more likely to be due to spread than to division of transduced neurons, because neurogenesis is mostly complete by embryonic day (E) 14.^12^ There appears to be a strong influence of age in determining cell tropism of AAV9, where injection at P0 (i.e., day of birth) results in predominately neuronal transduction,^14^ but injection at P2 or P3 causes a shift to higher astrocytic transduction.^12^ Our findings of largely neuronal transduction by both AAV9 and AAV-PHP.eB are in line with previous findings, but transduction differences caused by variation in post-natal day of injection should be considered if working with AAV-PHP.eB for this purpose.

We found no spread of either AAV vector beyond the olfactory bulb after intranasal injection. Although previous studies have shown that intranasal administration of viral vectors can produce global therapeutic effects in the brain, these were using transgenes that produce secreted products^19^ and/or utilized a longer course of delivery across multiple days or weeks.^20^^,^^21^ Neither GFP nor tdTomato are secreted proteins, and we administered only a single dose of vector, so this is likely to explain why we saw no transduction deeper in the brain.

As noted, we replicated previous findings^4^^,^^9^^,^^10^ that when administered via i.v. injection in C57BL/6J mice, AAV-PHP.eB has greater CNS transduction efficiency compared with AAV9, presumably because of AAV-PHP.eB’s improved ability at crossing the BBB. We also found some interesting region-specific patterns of transduction that may not be accounted for by BBB permeability. In the cortex, pyramidal cells in layer 5 were preferentially transduced over cells in other layers, by both AAV-PHP.eB and AAV9, and regardless of administration route, consistent with previous observations of AAV9 transduction.^22^ In the hippocampus, cells in the CA2 region were highly transduced by both vectors after i.c.v. administration and by AAV-PHP.eB after i.v. injection. This indicates pyramidal cells in this region have a propensity for taking up AAV, which may be determined by the complement of surface receptors expressed on these cells.^23^ Area CA2 in particular is known to have differential expression of at least 50 genes compared with neighboring areas CA1 and CA3,^24^ and some of these may be involved in rAAV binding and/or endocytic uptake. Furthermore, both the ssAAV-PHP.eB and scAAV-PHP.eB vectors gave remarkably high transduction of the olfactory bulb, which may prove to be of particular benefit in diseases afflicting the olfactory bulb.

We also made the discovery that the B6C3 mouse strain is CNS non-permissive, rendering AAV-PHP.eB no more effective than AAV9 after i.v. injection. Although we did not test for AAV-PHP.eB’s ability to transduce neuronal cells by a central administration method in B6C3 mice, based on previous studies,^8^^,^^10^ we predict our findings are due to the decreased ability of AAV-PHP.eB to pass through the BBB in B6C3 mice. Because the B6C3 is generated by a cross between a C57BL/6J and C3H/Hej mouse, which are CNS permissive and non-permissive, respectively, and CNS permissiveness has a codominant inheritance pattern,^8^ it is likely that B6C3 mice have the LY6A polymorphisms of the C3H/Hej mice.

The AAV-PHP.eB capsid packaged with a sc genome produced an overall increase in CNS cell transduction efficiency, as expected based on the increased efficiency of sc genomes in AAV9 and PHP.B vectors.^18^^,^^25^ This increase, however, was small and may not be biologically relevant when considering the percentage of cells that need to be transduced for a therapeutic effect. The minimal difference in transduction between the two vectors could be explained by the presence of the woodchuck hepatitis virus post-transcriptional regulatory element (WPRE) in the single-stranded plasmid, which has been shown to increase *in vivo* gene expression from AAV vectors up to 8-fold.^26^ One disadvantage to the sc genome is that it halves the rAAV packaging capacity compared with a single-stranded genome, restricting the size of the transgene to about 2.2 kb, which is limiting in terms of adding large regulatory elements, such as WPRE.^27^ Higher transduction by scAAV-PHP.eB may also be accounted for by an increased proportion of transgene expression in non-neuronal cells compared with the predominately neuronal cell transduction by ssAAV-PHP.eB. The present findings emphasize the relevance of genome configuration in respect to the gene therapy application, as a result of varying benefits from the use of single-stranded versus sc vectors dependent on the transgene size and desired cell targets.

Regardless of the administration route or animal age, AAV-PHP.eB showed benefit in the periphery in that it produced less off-target transduction of the liver than AAV9. The increased CNS specificity of AAV-PHP.eB is ideal for limiting unwanted off-target effects that may occur because of transgene expression in non-CNS organs, which can be toxic and trigger immune responses from the liver.^28^ Both vectors showed very low tropism for heart tissue, which would be unlikely to produce any significant off-target effects.

Although we did find a substantial increase in transduction from AAV-PHP.eB over AAV9, compared with the original report,^4^ the total number of cells transduced remained relatively low across all brain regions assessed, despite our use of a strong ubiquitous promoter. For example, we found 12% ± 2% of DAPI^+^ cortical cells were transduced, as compared with 51% ± 2% reported by the original authors.^4^ This difference could be accounted for by the site of i.v. injection, because Chan et al.^4^ delivered their injections into the retro-orbital sinus, the venous sinus located behind the eye. The proximity of this sinus to the brain could mean more of the vector is immediately accessible to the brain, before being circulated and cleared from the bloodstream, as compared with injection into the tail vein at the far side of the circulatory system. Indeed, it has previously been shown that injection of AAV9 in P1 pups into two different facial veins (retro-orbital and temporal facial vein) can produce dramatic differences in CNS transduction,^29^ with retro-orbital injection giving significantly higher transduction. This emphasizes the importance of the administration method in determining transduction outcome and is an area worth further exploration when considering translation of these techniques between animal models and to humans. Despite not replicating the remarkably high level of transduction found by Chan et al.,^4^ having a smaller number of transduced cells may still provide enough transgene expression to produce therapeutic effects, particularly in the case of transgenes that produce secreted proteins. The global transduction across cortical and subcortical structures produced by AAV-PHP.eB after i.v. administration is an important characteristic of this vector, given that many neurological diseases afflict cells across the whole brain.

Together, these data demonstrate the effectiveness of AAV-PHP.eB as a viral vector for a range of potential gene therapy applications and also highlight the limitations of using an *in vivo* evolution method given genetic variability within an animal species. In terms of feasibility from both a biological and a manufacturing standpoint, development of viral vectors that give good CNS transduction at low doses and with high specificity will be needed to allow for systemic administration of gene therapy in humans. However, the generation of AAV-PHP.eB has been an important proof-of-principle step in demonstrating that rAAV capsids can be modified to improve BBB permeability, and it will still prove valuable for preclinical testing despite enhanced CNS transduction not translating to all animal models.

## Materials and Methods

### AAV Vector Preparation

The ssAAV9.CB7.EGFP vector (4.0 × 10^13^ vg/mL) was produced by the Penn Vector Core (University of Pennsylvania, USA) using a pENN.AAV.CB7.CI.EGFP.WPRE.rBG (gift from James M. Wilson; Addgene #105542; http://addgene.org/105542; RRID: Addgene_105542) transgene plasmid and a pAAV2/9n (gift from James M. Wilson; Addgene #112865; http://addgene.org/112865; RRID: Addgene_112865) capsid plasmid. The ssPHP.eB.CAG.tdTomato vector (2.2 × 10^13^ vg/mL) was produced by Addgene (gift from Edward Boyden; Addgene viral prep #59462-PHPeB; http://addgene.org/59462; RRID: Addgene_59462; USA). The scPHP.eB.CAG.EGFP vector (1.22 × 10^13^ vg/mL) was produced by the Otago Viral Vector Facility (University of Otago, New Zealand, Enviromental Protection Agency (EPA) approval GMD101648) by standard triple-transfection methods into HEK293FT cells using a pscAAV-CAG-EGFP transgene plasmid (gift from Mark Kay; Addgene plasmid #83279; http://addgene.org/83279; RRID: Addgene_83279), a pUCmini-iCAP-PHP.eB (gift from Viviana Gradinaru; Addgene plasmid #103005; http://addgene.org/103005; RRID: Addgene_103005) capsid plasmid, and a pAdDeltaF6 helper plasmid (gift from James M. Wilson; Addgene plasmid #112867; http://addgene.org/112867; RRID: Addgene_112867). The vector was formulated in phosphate-buffered saline (PBS), and titer was determined by qPCR. CAG/CB7 are ubiquitous promoters and are of identical sequence across the three vectors in the present study.^30^

### Animals

Male wild-type C57BL/6J (n = 29) and B6;C3-Tg(APPswe,PSEN1dE9)85Dbo/Mmjax (B6C3) (n = 4) mice were maintained as colonies at the University of Otago, New Zealand. Animals treated at 3 months were group housed in standard caging until treatment and were transferred to single housing after treatment to prevent injury from fighting. Animals treated at P1 were housed with the dam until weaning at 21 days. Food and water were available *ad libitum*, and the cage contained shredded paper bedding as standard housing. Animals were kept on a 12-h light/dark cycle (lights on at 6:00 h), and the room temperature was controlled via a thermostat set at 21°C. All procedures were approved by the University of Otago Animal Ethics Committee and conducted in accordance with New Zealand Animal Welfare and Biosecurity Legislation.

### Vector Administration

#### Tail-Vein Injection

Three-month-old C57BL/6J (n = 9) and B6C3 mice (n = 4) were injected (100 μL total volume) in the lateral tail vein with a dose of 1 × 10^11^ vg/mouse each of ssAAV9-EGFP and ssPHP.eB-tdTomato. Further, 3-month-old C57BL/6J mice (n = 6) were injected (100 μL total volume) in the lateral tail vein with a dose of 1 × 10^11^ vg/mouse each of ssPHP.eB-tdTomato and scPHP.eB-GFP. Mice were anesthetized with isoflurane gas (2%, 200 mL/min), and vectors were combined and diluted in sterile saline to reach the final injection volume.

#### Intranasal Injection

Three-month-old C57BL/6J mice (n = 6) were administered a dose of 1 × 10^11^ vg/mouse each of ssAAV9-EGFP and ssPHP.eB-tdTomato (7 μL total volume) in the left naris. Mice were anesthetized with isoflurane gas (2%, 200 mL/min), and the vectors were combined before administration. The vectors were administered by applying a series of 1-μL drops at 10-s intervals with a micropipette to the nasal cavity of each mouse while the mouse was held in an inverted position.

#### i.c.v. Injection

Three-month-old C57BL/6J mice (n = 4) were anesthetized with isoflurane gas (2%, 200 mL/min). After mice were immobilized in a stereotaxic apparatus, a linear skin incision was made over the midline, and a 1-mm burr hole was drilled in the skull 0.4 mm anterior and 1 mm lateral to the midline on the right hemisphere using a mechanical drill. ssAAV9-EGFP and ssPHP.eB-tdTomato (1 × 10^10^ vg of each vector) were combined, then diluted to a final volume of 2 μL before administration. Vectors were injected unilaterally into the lateral ventricle 1.65 mm below the surface of the skull using a Hamilton syringe mounted to a microinjection pump with a 33G needle. Injection rate was 150 nL/min. The needle remained in place in the injection site for an additional 10 min before being removed. Animals were administered carprofen (5 mg/kg) and lidocaine (4 mg/kg) for analgesia.

P1 C57BL/6J pups (n = 4) were cryoanesthetized on a paper towel on wet ice for 6 min until no pedal withdrawal reflex was observed. i.c.v. injections were conducted using a 1-mL insulin syringe with a pulled glass capillary needle tip (1-μm diameter). ssAAV9-EGFP and ssPHP.eB-tdTomato (1 × 10^10^ vg of each vector) were combined and diluted to a final volume of 4 μL before administration. Vectors were injected into the left hemisphere 2 mm lateral to the midline, midway between bregma and lambda, and to a depth of 1 mm below the surface of the skull.^31^ Following injection, pups were placed on a warming blanket and returned to the dam in their own cage once they were visibly active. Pups were monitored for 4 days following injection and were weaned at 21 days.

### Histological Processing

Thirty days post-injection, mice were deeply anesthetized with pentobarbital (200 mg/kg) and then transcardially perfused with 20 mL of 0.01 M PBS (pH 7.2) followed by 20 mL of fixation solution containing 4% paraformaldehyde in 0.1 M phosphate buffer (PB). Brain, liver, and heart tissue were dissected and then fixed in 4% paraformaldehyde in PB for 1 day. Organs were then transferred to 30% sucrose in PB and allowed to sink to the bottom of the solution (2 days). Serial sagittal sections of the entire brain (40 μm thickness), liver (20 μm thickness), and heart (20 μm thickness) were cut on a freezing microtome (HM325 NX; Thermo Scientific). Sections were then stored in cryoprotectant solution containing 30% sucrose and 30% ethylene glycol in PB at −20°C until further use.

Brain sections were stained as free-floating sections in 12-well plates. Sections were washed three times in PBS before incubation for 1 h at room temperature in a blocking solution containing 0.1% Triton X-100 and 10% normal goat serum (v/v). Primary antibody labeling was done overnight at 4°C. The antibody used in this study was guinea pig anti-NeuN (266-004, RRID: AB_2619988; Synaptic Systems, Germany) (at 1:500).

Brain sections were then washed four times in PBS before incubation with the appropriate secondary antibodies in blocking solution at room temperature for 2 h. The secondary antibody used was Alexa Fluor 647 anti-guinea pig IgG (A21450, RRID: AB_2735091; Thermo Fisher) (at 1:500). Sections were then washed again as above before mounting on glass slides with antifade solution containing DAPI nuclei acid stain (RRID: AB_2629482; Invitrogen, Eugene, OR, USA).

Liver and heart sections were mounted on glass slides with antifade solution containing DAPI nuclei acid stain (RRID: AB_2629482; Invitrogen, Eugene, OR, USA).

For manual counting of double-positive cells, images were taken on a confocal microscope (Nikon C2+) with a 40× objective lens. For whole-brain images, slides were imaged using a Cytation 5 Multi-Mode Reader (BioTek).

### Statistical Analyses

Data were compiled in Microsoft Excel (2016), and statistical analyses were carried out in GraphPad Prism (8.2.1). Cell counts from either whole-brain regions or multiple regions of interest from three to four sections for each animal were combined and then compared between conditions. Data are expressed as mean ± SEM. Differences between vectors were analyzed using paired or unpaired Student’s t tests. The p values <0.05 were considered statistically significant.

## Author Contributions

Study Design: S.N.M., J.L.L., L.S., W.C.A., S.M.H. AAV Delivery Training: L.S. Conducted Experiments: S.N.M., J.L.L., L.S. Analyzed the Data: S.N.M., J.L.L. Writing – Original Draft: S.N.M. Writing – Review & Editing: S.N.M., J.L.L., L.S., W.C.A., S.M.H.

## Conflicts of Interest

The authors declare no competing interests.
